# Preoperative Staging in Breast Cancer: Intraindividual Comparison of Unenhanced MRI Combined With Digital Breast Tomosynthesis and Dynamic Contrast Enhanced-MRI

**DOI:** 10.3389/fonc.2021.661945

**Published:** 2021-05-04

**Authors:** Veronica Rizzo, Giuliana Moffa, Endi Kripa, Claudia Caramanico, Federica Pediconi, Francesca Galati

**Affiliations:** Department of Radiological, Oncological and Pathological Sciences, Sapienza University of Rome, Rome, Italy

**Keywords:** breast cancer, preoperative staging, digital breast tomosynthesis, unenhanced protocol, diffusion weighed imaging

## Abstract

**Objectives:**

To evaluate the accuracy in lesion detection and size assessment of Unenhanced Magnetic Resonance Imaging combined with Digital Breast Tomosynthesis (UE-MRI+DBT) and Dynamic Contrast Enhanced Magnetic Resonance Imaging (DCE-MRI), in women with known breast cancer.

**Methods:**

A retrospective analysis was performed on 84 patients with histological diagnosis of breast cancer, who underwent MRI on a 3T scanner and DBT over 2018-2019, in our Institution. Two radiologists, with 15 and 7 years of experience in breast imaging respectively, reviewed DCE-MRI and UE-MRI (including DWI and T2-w) + DBT images in separate reading sections, unaware of the final histological examination. DCE-MRI and UE-MRI+DBT sensitivity, positive predictive value (PPV) and accuracy were calculated, using histology as the gold standard. Spearman correlation and regression analyses were performed to evaluate lesion size agreement between DCE-MRI *vs* Histology, UE-MRI+DBT *vs* Histology, and DCE-MRI *vs* UE-MRI+DBT. Inter-reader agreement was evaluated using Cohen’s κ coefficient. McNemar test was used to identify differences in terms of detection rate between the two methodological approaches. Spearman’s correlation analysis was also performed to evaluate the correlation between ADC values and histological features.

**Results:**

109 lesions were confirmed on histological examination. DCE-MRI showed high sensitivity (100% Reader 1, 98% Reader 2), good PPV (89% Reader 1, 90% Reader 2) and accuracy (90% for both readers). UE-MRI+DBT showed 97% sensitivity, 91% PPV and 92% accuracy, for both readers. Lesion size Spearman coefficient were 0.94 (Reader 1) and 0.91 (Reader 2) for DCE-MRI *vs* Histology; 0.91 (Reader 1) and 0.90 (Reader 2) for UE-MRI+DBT *vs* Histology (p-value <0.001). DCE-MRI *vs* UE-MRI+DBT regression coefficient was 0.96 for Reader 1 and 0.94 for Reader 2. Inter-reader agreement was 0.79 for DCE-MRI and 0.94 for UE-MRI+DBT. McNemar test did not show a statistically significant difference between DCE-MRI and UE-MRI+DBT (McNemar test p-value >0.05). Spearman analyses showed an inverse correlation between ADC values and histological grade (p-value <0.001).

**Conclusions:**

DCE-MRI was the most sensitive imaging technique in breast cancer preoperative staging. However, UE-MRI+DBT demonstrated good sensitivity and accuracy in lesion detection and tumor size assessment. Thus, UE-MRI could be a valid alternative when patients have already performed DBT.

## Introduction

Breast cancer is the most common female invasive cancer and the most frequent cause of cancer death in women, worldwide (1). Formerly, the standard modalities for breast cancer diagnosis and preoperative staging included conventional imaging (full-field digital mammography - FFDM, and breast ultrasonography - US), and percutaneous image-guided biopsy. To date, the value of dynamic contrast-enhanced magnetic resonance imaging (DCE-MRI) as the most sensitive technique in breast imaging is well established. Considering the limitations of conventional breast imaging, several studies have progressively promoted the role of DCE-MRI for breast cancer staging. However, the unrestricted use of preoperative breast MRI remains controversial (2), since only certain subgroups of patients benefit from presurgical staging with breast MRI (3, 4). Furthermore, DCE-MRI has some main disadvantages, including variable specificity, need of contrast agent administration (with the well-known associated risks, such as adverse reactions, brain deposition, and nephrogenic systemic fibrosis in patients with terminal renal insufficiency), long exam time and high costs. To overcome these disadvantages, unenhanced MRI (UE-MRI) and abbreviated protocols have been developed and recently are starting to be implemented into clinical practice (5).

UE-MRI is based on diffusion weighted imaging (DWI) (or DWI combined with T1 and/or T2-weighted sequences). DWI measures the water diffusivity of the tissues under examination, that can be quantitatively assessed using the apparent diffusion coefficient (ADC) value. Lower ADC values are associated with high cellular density, typical of malignant tissues. As a consequence, DWI represents a valuable tool to distinguish benign from malignant breast lesions, showing a higher specificity (75-84%) than DCE-MRI (67-72%) (6, 7). Other advantages of this approach are cost and time saving and the extension of MRI feasibility to patients not suitable for contrast agent administration.

Digital breast tomosynthesis (DBT) is a fast, highly available technique, which provides a 3D reconstruction of the breast, improving lesion detection, characterization and localization (8–11) and reducing both false negatives and false positives rates (12). DBT is cheaper than MRI and devoid of significant contraindications, despite keeping a high accuracy in the detection of breast cancer and a high sensitivity in tumor size assessment, both when evaluated alone (13) and in addition to US and/or mammography (14–17). As a consequence, DBT has been taken into consideration as a valid alternative modality in case of contraindications to MRI.

Assuming that the combination of UE-MRI and DBT could emphasize the advantages of both techniques, as far as we know, there is only one study (18) that has compared the diagnostic accuracy of UE-MRI combined with DBT to DCE-MRI, in the preoperative setting, and it was performed on a 1.5 T scanner and included a relatively small population.

On these premises, the main objective of this study was to evaluate the accuracy of preoperative UE-MRI combined with DBT (UE-MRI+DBT) compared to DCE-MRI, on a 3T scanner, firstly with regard to lesion size assessment and secondly in terms of lesion detection. As a minor purpose, the study aimed to investigate possible correlations between ADC values and histological features.

## Materials and Methods

This study was conducted according to Good Clinical Practice guidelines and obtained the approval of our institutional review board. The requirement for informed consent was waived because of the retrospective nature of the study. Patient data were acquired anonymously, using the institutional database.

All the women with a new biopsy-proven diagnosis of breast cancer who underwent DBT and preoperative breast MRI (according to our Breast Unit multidisciplinary team indications) within one month, from March 2018 to December 2019, were considered for this study.

Definitive breast surgery, including lumpectomy, quadrantectomy and mono- or bilateral mastectomy, was performed in all patients less than 1 month after DBT and MRI examinations.

### DBT Examination

DBT was performed on a dedicated FFDM system (MAMMOMAT Inspiration; Siemens AG Healthcare, Erlangen - Germany).

Bilateral mediolateral oblique (MLO) views were acquired for all patients. Craniocaudal (CC) projection of the affected side was added in 22 cases, since it allowed a better visualization of a specific lesion.

Images were reconstructed using the filtered back-projection algorithm, in order to provide sections parallel to the breast support. DBT reconstructions (section thickness = 1 mm) were evaluated in automatic or manual scroll modes on a dedicated workstation using two 5-Megapixel diagnostic monitors (Nio 5MP MDNG-6121; Barco NV, Kortrijk - Belgium).

### MRI Examination

All bilateral breast MRI examinations were performed on a 3 T scanner (Discovery MR 750; GE Healthcare, Chicago - IL, USA) using a dedicated 8-channel surface coil and patients in a prone position.

The minimum MRI protocol considered valid for the study included: axial pre-contrast 2D fast spin echo T2-weighted fat-suppressed sequences (*repetition time [RT] = 11,000 ms, echo time [ET] = 119 ms, echo train length [ETL] = 19, bandwidth = 62.50 kHz, matrix = 512×224, thickness = 4 mm, interval = 0.1, field of view [FOV] = 350×350 mm, number of excitations [NEX] = 1, scan time = 132 s*), axial pre-contrast diffusion-weighted echo-planar imaging (DWI-EPI) sequences (*RT=4983 ms, ET=58 ms, bandwidth = 250 kHz, matrix = 150×150, thickness = 4 mm, FOV = 350×350 mm, NEX = 2–2-4, scan time = 229 s, with b values of 0, 500 and 1000 s/mm^2^*), axial 3D spoiled gradient-echo T1-weighted fat-suppressed sequences (*flip angle = 15°, RT = 4 ms, ET = 2 ms, bandwidth = 166.67 kHz, matrix = 320×320, thickness = 1.40 mm, FOV = 340×340 mm, NEX =1*) acquired one time before and nine times after contrast agent administration (*total scan time = 363 s*), and sagittal post-contrast 3D spoiled gradient-echo T1-weighted fat-suppressed sequences.

Fat suppression of T2-weighted sequences was based on a three-point Dixon technique (IDEAL). ADC maps were calculated automatically. Subtraction images were obtained in post-processing for all examinations. The total acquisition time was about 13 minutes.

A dose of 0.1 mmol/kg (0.2 mL/kg) gadoteridol (Prohance 279.3 mg/mL; Bracco Imaging SpA, Milano - Italy) was power-injected through a peripheral venous access (22 gauge) at a rate of 3 mL/sec and was followed by a 20-mL saline flush at the same rate.

Imaging of pre-menopausal women was performed between the 7th and 14th day of the menstrual cycle, according to current guidelines (19).

### Imaging Evaluation

Two dedicated breast radiologists, with 15 and 7 years of experience respectively, randomly evaluated DBT and MR imaging sets at a dedicated workstation and classified the detected lesions according to the 2013 American College of Radiology Breast Imaging Reporting and Data System (ACR BI-RADS) lexicon (20).

The readers were blinded to the study design and to clinical and histopathological information (including the presence and the benign or malignant nature of the lesions, their position and size).

The two readers first evaluated DBT combined with UE-MRI sequences (T2-weigthed sequences and DWI with corresponding ADC maps).

All suspicious lesions (index and additional lesions) detected were measured and included in the statistical analysis, both for the detection rate and for the tumor size assessment.

In order to obtain the most reliable tumor size, the readers measured the maximum diameter of each lesion on both DBT and UE-MRI. The lesion sizes were considered to be concordant when they showed a difference of less than 3 mm. In cases of conflicting results of DBT and UE-MRI assessments, readers chose the imaging modality where the lesion was better recognizable. Size in mm was reported.

Masses were assessed measuring only tumor’s core, to avoid possible overestimation due to the desmoplastic reaction surrounding the lesion. Non-mass lesions on UE-MRI and calcifications and architectural distortion on DBT were measured acquiring their maximum extent.

Hyperintensity on DWI was assessed qualitatively using high b-value images (b = 1000 s/mm^2^). Subsequently, ADC values of the areas of restricted diffusion were obtained automatically after drawing manually a 2D circular region of interest with an area at least of 5 mm^2^.

DCE-MR imaging sets were randomly evaluated on average two weeks after UE-MRI+DBT reading, to avoid possible recall bias. Shape and margins (for masses), distribution (for non-mass enhancements) and internal enhancement characteristics were assessed on post-contrast fat-suppressed T1-weighted sequences and reported.

Moreover, each lesion was measured again on the slice where it appeared largest. All suspicious lesions detected (index and additional lesions) were included in the statistical analysis.

### Histopathological Analysis

Histopathological analyses were performed according to standardized protocols by a pathologist with more than 20 years of experience. Tumor size was measured on the surgical specimen for each lesion and considered as the gold standard.

Tumors were classified following the World Health Organization Classification and graded according to the Nottingham Histologic Score.

On the basis of immunohistochemical features, including the expression of estrogen and progesterone receptors, the expression of human epidermal growth factor receptor 2 (HER2), and the assessment of the Ki-67 proliferation index, invasive tumors were classified as luminal A-like, luminal B-like, HER2-positive and triple negative, according to the 2013 St. Gallen International Breast Cancer Conference classification (21).

### Statistical Analysis

Sensitivity, positive predictive value (PPV) and accuracy were calculated for both DCE-MRI and UE-MRI+DBT to investigate lesion detection rates, using histology as the gold standard.

Spearman’s correlation coefficient and regression analyses were fitted to the positive cases and were used to evaluate DCE-MRI and UE-MRI+DBT measurements agreement and their dependence, with respect to the histological results.

The measurements were considered concordant with histology if there was within ± 3 mm difference compared to the gold standard (consequently, there was underestimation if the difference was <3 mm and overestimation if it was >3 mm).

Separate regression analyses were used to investigate the effect of mass and non-mass enhancements at DCE-MRI on lesion size measurement.

Spearman’s correlation analysis was also performed to evaluate the correlation between ADC values, histological lesion grade and other variables.

Each analysis was performed separately for both readers (Reader 1 and Reader 2). Inter-reader agreement was analyzed using Cohen’s κ coefficient.

McNemar test was used to evaluate differences between DCE-MRI and UE-MRI+DBT in terms of lesion detection rate.

P-values <0.05 were considered statistically significant.

Statistical analyses and graphs plotting were realized using IBM SPSS Statistics software, version 25.

## Results

**Figure 1:**
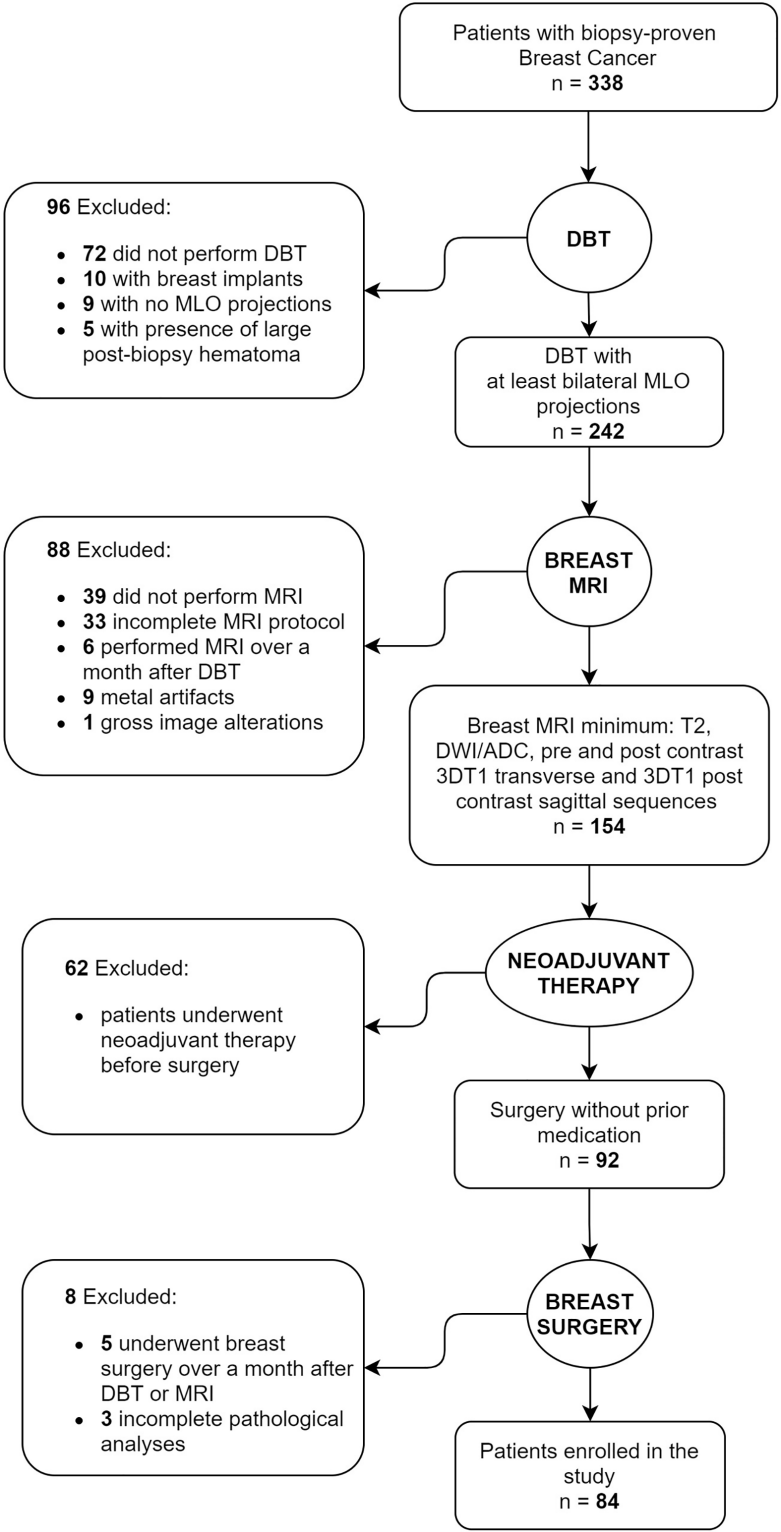
Flowchart of patient enrollment and exclusion criteria.

Of a total of 338 patients initially identified, the final study population included 84 women (mean age 55.6 years; range 34-83 years). Exclusion flowchart is shown in **Figure 1**.

**Figure 2:**
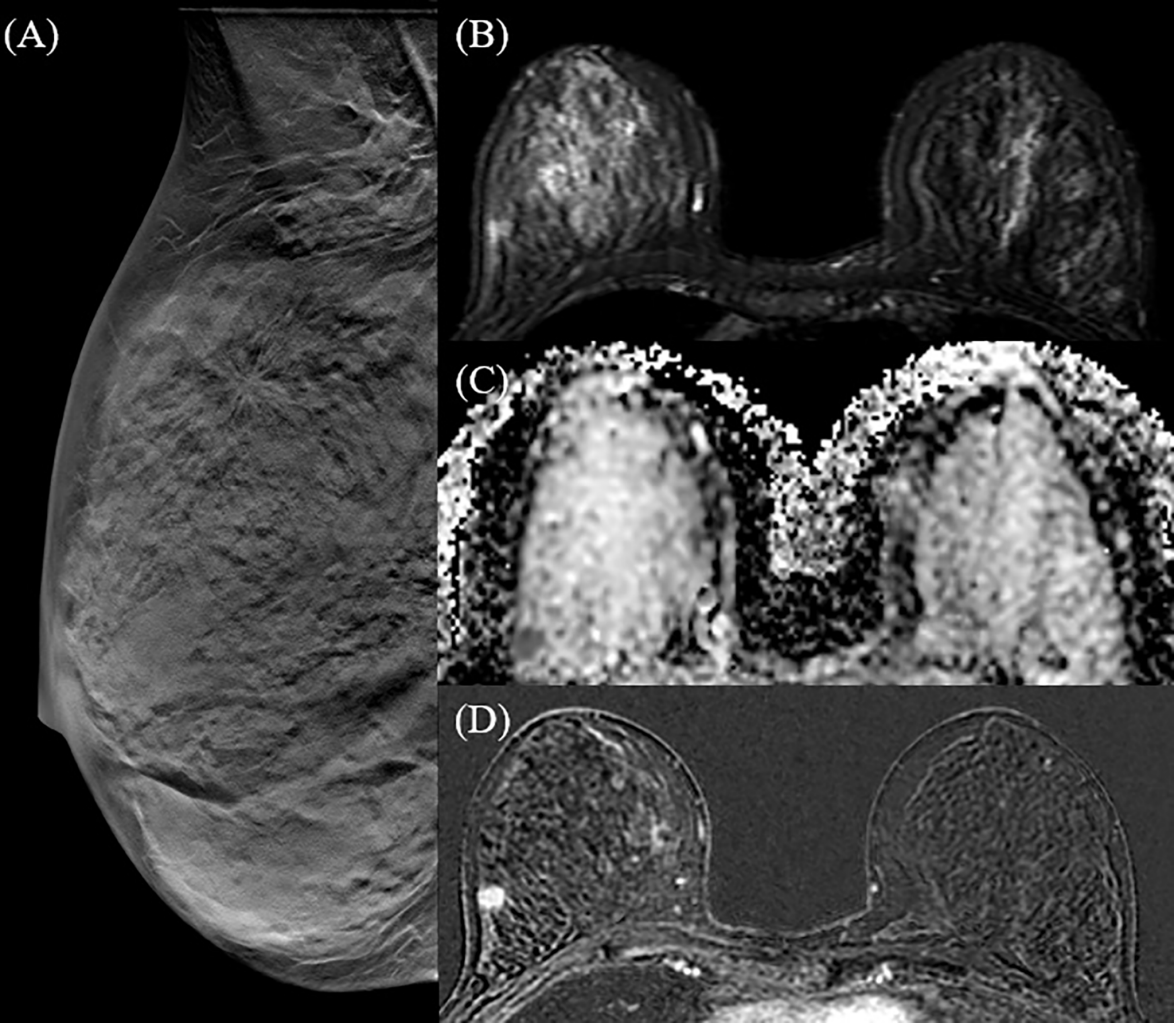
42 y-o woman with unifocal invasive ductal carcinoma (IDC). Right breast DBT MLO projection **(A)** identifies a single spiculated opacity in the upper-outer quadrant, corresponding to the hyperintense area in the T2 fat-saturated sequence **(B)** and to the hypointense area in the ADC map **(C)**. T1 fat-saturated post-contrast post-processing subtraction image **(D)** confirms a mass enhancement in the right breast, with high morphological and dimensional correspondence.

**Figure 3:**
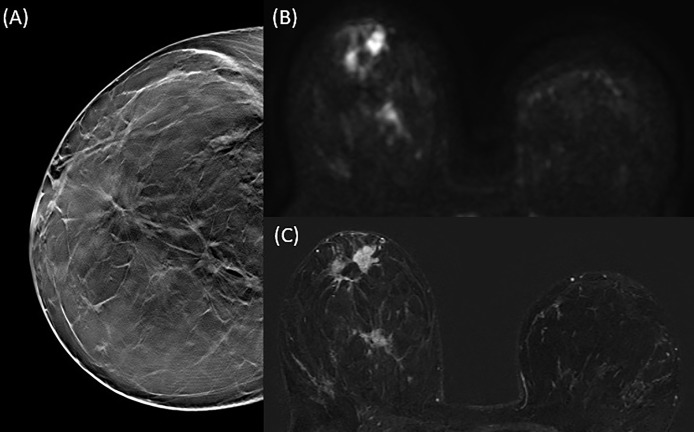
50 y-o woman with multicentric disease (IDC). DBT CC projection **(A)** clearly identifies three parenchymal distortions in the right breast corresponding to three spiculated hyperintense areas on DWI **(B)** and three areas of mass enhancement on T1 fat-saturated contrast-enhanced post-processing subtraction image **(C)**, with a good correlation in terms of lesions number and size.

Breast cancer was unifocal in 51 patients (**Figure 2**), multifocal in 18, multicentric in 13 (**Figure 3**) and bilateral in 2 patients, for a total of 109 malignant breast lesions histologically confirmed. Mean and median lesion size measured on the surgical specimen were 21.9 mm (SD = 15.6 mm) and 16 mm, respectively. According to the World Health Organization Classification the malignant lesions identified included 68 invasive ductal carcinomas (IDC), 15 invasive ductal carcinomas with foci of ductal carcinoma *in situ* (IDC + DCIS foci), 16 ductal carcinomas *in situ* (DCIS), 6 invasive lobular carcinomas (ILC), 2 papillary carcinomas, and 2 mucinous carcinomas. The lesions histologically classified as IDC, IDC + DCIS foci and ILC were further differentiated according to the 2013 St. Gallen International Breast Cancer Conference classification in luminal A (n = 39), luminal B (n = 40), HER2-positive (n = 4), and triple negatives (n = 6).

### UE-MRI+DBT

#### Lesion Detection

Both readers found 117 lesions reading DBT and UE-MRI; 106 were true positive lesions, while 11 detected lesions did not correspond to real malignant lesions at pathological analyses and were classified as false positives. 3 lesions were not visible and considered as false negative findings.

Sensitivity, PPV and accuracy were 97%, 91% and 92%, respectively, for both readers.

#### Tumor Size Assessment

Regarding lesions size, concordance with histopathology was achieved for 77 and 78 lesions, respectively by the two readers, 15 and 12 cases were underestimated while 14 and 16 were overestimated (**Table 1**).

**Table 1:** Concordance in terms of lesion size assessment of the malignant lesions detected with UE-MRI+DBT and histology.

Histology	N. of lesions	*UE-MRI+DBT*								
		Reader 1				Reader 2			
		Overestimated	Agreement	Underestimated	Negatives	Overestimated	Agreement	Underestimated	Negatives
DCIS	16 (14,7%)	3 (18,75%)	11 (68,75%)	2 (12,5%)	0 (0%)	2 (12,5%)	12 (75%)	2 (12,5%)	0 (0%)
IDC	68 (62,4%)	5 (7,35%)	51 (75%)	10 (14,7%)	2 (2,95%)	7 (10,3%)	52 (76,5%)	7 (10,3%)	2 (2,9%)
IDC+DCIS foci	15 (13,8%)	5 (33,3%)	9 (60%)	1 (6,7%)	0 (0%)	5 (33,3%)	9 (60%)	1 (6,7%)	0 (0%)
ILC	6 (5,5%)	0 (0%)	4 (66,7%)	2 (33,3%)	0 (0%)	1 (16,7%)	3 (50%)	2 (33,3%)	0 (0%)
MUCINOUS C.	2 (1,8%)	1 (50%)	1 (50%)	0 (0%)	0 (0%)	1 (50%)	1 (50%)	0 (0%)	0 (0%)
PAPILLARY C.	2 (1,8%)	0 (0%)	1 (50%)	0 (0%)	1 (50%)	0 (0%)	1 (50%)	0 (0%)	1 (50%)
**Total**	**109 (100%)**	**14 (12,8%)**	**77 (70,6%)**	**15 (13,8%)**	**3 (2,8%)**	**16 (14,7%)**	**78 (71,5%)**	**12 (11%)**	**3 (2,8%)**

Mean lesion size was 21.9 mm (SD = 15.6 mm) for Reader 1 and 22.3 mm (SD = 15.7 mm) for Reader 2.

Spearman correlation coefficient for lesion size (UE-MRI+DBT *vs* Gold standard) was 0.91 for Reader 1 and 0.90 for Reader 2 (p-value <0.001).

**Figure 4:**
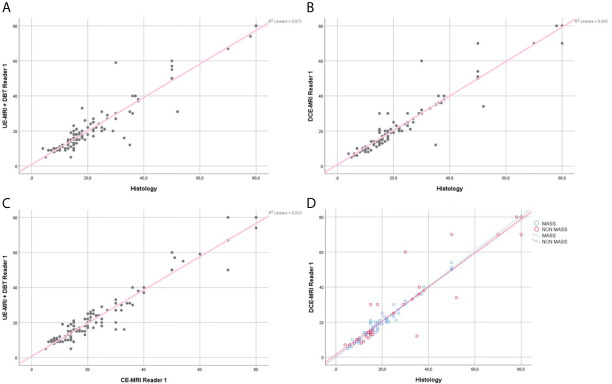
Reader 1 lesions size assessment. Regression analysis for the lesion size, comparing UE-MRI+DBT *vs* Histology **(A)**, DCE-MRI *vs* Histology **(B)** and UE-MRI+DBT *vs* DCE-MRI **(C)**. Section **(D)** shows the regression analysis performed dividing the lesions into the mass and non-mass groups, comparing DCE-MRI *vs* Histology.

**Figure 5:**
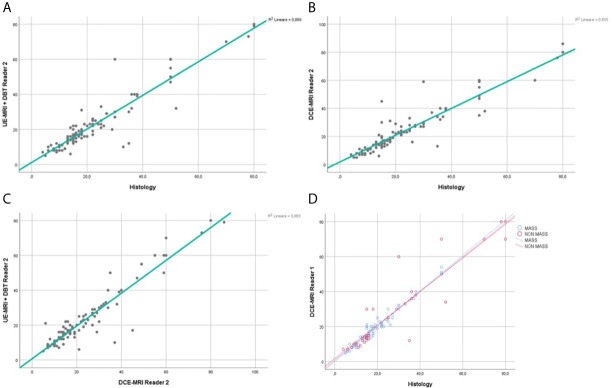
Reader 2 lesions size assessment. Regression analysis for the lesion size, comparing UE-MRI + DBT *vs* Histology **(A)**, DCE-MRI *vs* Histology **(B)** and UE-MRI+DBT *vs* DCE-MRI **(C)**. Section **(D)** shows the regression analysis performed dividing the lesions into the mass and non-mass groups, comparing DCE-MRI *vs* Histology.

The regression coefficient for the model where UE-MRI+DBT lesion size was the dependent variable and gold standard was the independent variable was equal to 0.93 (R-squared =0.87) for both readers (**Figures 4A**, **5A**).

The inter-reader agreement evaluated with Cohen’s κ was excellent: 0.94 (p <0.001).

#### Correlation Between ADC Values and Histological Features

**Figure 6:**
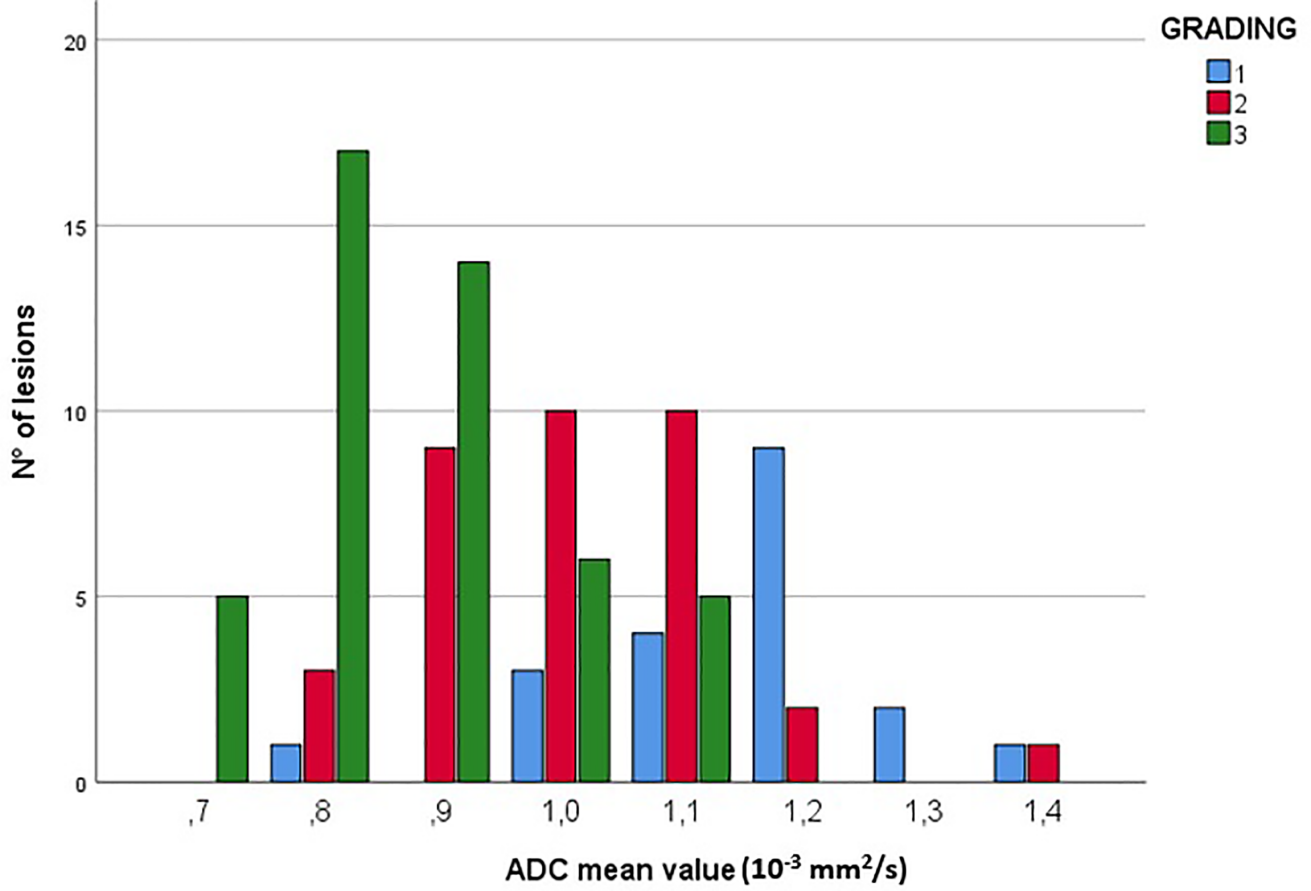
Correlation between ADC values (10^-3^mm^2^/s) and lesion grading. The figure shows the prevalence of low ADC values (≤1.1x10^-3^mm^2^/s) in G2 and G3 lesions.

For what concerns the correlation between DWI and histological features of the lesions, no statistically significant association was found between ADC values and specific subtypes of breast cancer (luminal A, luminal B, HER2-positive, and triple negatives), but there was a high prevalence of low ADC values (equal or less than 1.1x10^-3^mm^2^/s) in G2 and G3 lesions, with a decrease of ADC mean values as the histological grade increases (Correlation’s coefficient = -0.647; p-value <0.001) (**Figure 6**).

### DCE-MRI

#### Lesion Detection

Reader 1 detected 122 lesions, 13 of them were false positive findings, while 109 were true positives.

Reader 2 found 119 lesions, 12 of them did not correspond to real malignant lesions at pathological analyses and 107 lesions were true positives. 2 lesions were not detected.

Sensitivity, PPV and accuracy were 100%, 89% and 90% respectively for Reader 1, and 98%, 90% and 90% for Reader 2.

#### Tumor Size Assessment

Regarding lesions size, Reader 1 underestimated 4 lesions and overestimated 11 lesions, while 94 lesions were concordant with histological size. Reader 2, instead, underestimated 7, overestimated 18 and reached concordance in 82 cases (**Table 2**).

**Table 2:** Concordance in terms of lesion size assessment of the malignant lesions detected with DCE-MRI and histology.

Histology	N. of lesions	*DCE-MRI*								
		Reader 1				Reader 2			
		Overestimated	Agreement	Underestimated	Negatives	Overestimated	Agreement	Underestimated	Negatives
DCIS	16 (14,7%)	3 (18,75%)	13 (81,25%)	0 (0%)	0 (0%)	2 (12,5%)	12 (75%)	2 (12,5%)	0 (0%)
IDC	68 (62,4%)	3 (4,4%)	63 (92,7%)	2 (2,9%)	0 (0%)	8 (11,8%)	57 (83,8%)	2 (2,9%)	1 (1,5%)
IDC+DCIS foci	15 (13,8%)	4 (26,7%)	11 (73,3%)	0 (0%)	0 (0%)	6 (40%)	8 (53,3%)	1 (6,7%)	0 (0%)
ILC	6 (5,5%)	0 (0%)	4 (66,7%)	2 (33,3%)	0 (0%)	1 (16,7%)	3 (50%)	2 (33,3%)	0 (0%)
MUCINOUS C.	2 (1,8%)	1 (50%)	1 (50%)	0 (0%)	0 (0%)	1 (50%)	1 (50%)	0 (0%)	0 (0%)
PAPILLARY C.	2 (1,8%)	0 (0%)	2 (100%)	0 (0%)	0 (0%)	0 (0%)	1 (50%)	0 (0%)	1 (50%)
**Total**	**109 (100%)**	**11 (10,1%)**	**94 (86,2%)**	**4 (3,7%)**	**0 (0%)**	**18 (16,5%)**	**82 (75,2%)**	**7 (6,5%)**	**2 (1,8%)**

Mean lesion size was 21.8 mm (SD = 16.1 mm) for Reader 1 and 22.6 (SD = 15.9) for Reader 2.

Spearman correlation coefficient for lesion size (DCE-MRI *vs* Histology) was 0.94 for Reader 1 and 0.91 for Reader 2 (p-value <0.001).

The regression coefficient for the model where lesion size at DCE-MRI was the dependent variable and the gold standard was the independent variable was equal to 0.95 (R-squared = 0.89) for Reader 1 and 0.92 (R-squared = 0.85) for Reader 2 (**Figures 4B**, **5B**). The inter-reader agreement evaluated with Cohen’s κ was 0.79 (p <0.001).

In a separate regression model, where the lesions were divided into masses and non-mass enhancements, there was no significant difference in terms of correlation coefficients for lesion size for both readers (**Figures 4D**, **5D**), however non-mass lesions showed the tendency to have a slightly lower correlation compared to the mass group.

### UE-MRI+DBT *vs* DCE-MRI

#### Lesion Detection

Reader 1 detected 117 lesions and missed 1 lesion both in DCE-MRI and UE-MRI+DBT; 4 lesions were recognized only in DCE-MRI, while no lesion was identified only in UE-MRI+DBT.

Reader 2 detected 116 lesions and missed 2 lesions both in DCE-MRI and UE-MRI+DBT; 3 lesions were recognized only in DCE-MRI and 1 lesion was detected only in UE-MRI+DBT.

The difference in detection rate between DCE-MRI and UE-MRI+DBT was not statistically significant (McNemar test p-value >0.05 for both readers).

#### Tumor Size Assessment

The regression coefficient for the model where UE-MRI+DBT lesion size was the dependent variable while DCE-MRI measure was the independent variable was 0.96 (R-squared = 0.92) for Reader 1 and 0.94 (R-squared = 0.88) for Reader 2 (**Figures 4C**, **5C**).

## Discussion

The goals of preoperative staging of breast cancer are the size assessment of the index lesion and the search for further ipsilateral or contralateral lesions. Clearly, accuracy is fundamental in such an evaluation, since it can influence future treatment. Despite several studies have progressively promoted the role of breast MRI to complete conventional imaging for preoperative staging, due to evidence-based benefits, the limited number of randomized clinical trials (22–26) that evaluated the impact of preoperative MRI on treatment regimens and surgical outcomes have given conflicting results. A large-scale observational multicenter prospective study (MIPA study) was launched internationally in 2012 but is currently at the analytic phase (27). As a consequence, the role of preoperative breast MRI remains controversial for now, and alternative imaging modalities have been considered to evaluate the extent of disease during the preoperative planning. In particular, a few Authors have evaluated the role of DBT compared to MRI in preoperative staging, directly (13) or, in more studies, combined with US and/or mammography (14–17). The results of these studies have confirmed that DCE-MRI is the most accurate imaging technique in the preoperative staging of breast cancer, even if DBT has shown a good diagnostic performance.

Furthermore, the recent concern about the risks associated with gadolinium-based contrast agents and the recommendation to limit their use to when strictly necessary (28) have encouraged the evaluation of UE-MRI protocols for the detection of breast cancer, reaching good specificity (90%) and acceptable sensitivity (76-78%) (29). UE-MRI protocols are built mainly on DWI, that has the advantage of increasing MRI specificity (30). In addition, it was demonstrated that T2-weighted sequences increase the specificity in differential diagnosis between benign and malignant lesions (31) and that T2-weighted imaging combined with DWI allow a good diagnostic performance (29).

Baltzer et al. (32) have suggested that UE-MRI using DWI only may represent a valid alternative to DCE-MRI, as both provide comparable results. However, Pinker et al. (30) have subsequently argued that DWI is inadequate as a stand-alone parameter for breast cancer detection. The main problem with DWI is the still limited spatial resolution (30). Consequently, it is reasonable to assume that the combination of UE-MRI, based on T2-weigthed and DWI sequences, with a high-spatial resolution technique such as DBT, could counterbalance the lack of spatial resolution of DWI, ensuring a high-contrast resolution.

Therefore, the aim of this study was to evaluate the accuracy of preoperative UE-MRI combined with DBT in terms of tumor size assessment and detection of additional ipsilateral or contralateral lesions, compared to DCE-MRI, on a 3 T scanner.

The results of our study have confirmed that, independently of the radiologist experience, DCE-MRI is the most sensitive technique in lesion detection, even though UE-MRI+DBT showed a good sensitivity as well. PPV was slightly higher in UE-MRI+DBT than in DCE-MRI and also the accuracy was better in the first diagnostic method. Interestingly, UE-MRI+DBT provided a lower number of false positives than DCE-MRI, although the difference was minimal. However, both readers found less false negative cases evaluating DCE-MRI.

Our results are consistent with that of the aforementioned similar study by Girometti et al. (18) on a cohort of 56 women with breast cancer. They concluded that UE-MRI+DBT has shown comparable sensitivity (85.7-88.6%) than DCE-MRI (94.3–100%), with significant differences depending on the reader’s experience.

Regarding tumor size assessment, our correlation analyses demonstrated a strong agreement between both UE-MRI+DBT and DCE-MRI results and pathology, even if Spearman correlation coefficient was higher for DCE-MRI than UE-MRI+DBT. These results were similar to what previously reported by Mariscotti et al. (14) who have compared FFDM, DBT, US, MRI and their combination in the preoperative setting, concluding that DCE-MRI has shown the strongest agreement with histology. In contrast with these evidences and our results, Girometti et al. (33) have found that UE-MRI alone showed the greatest concordance with pathology in terms of cancer size, even compared to DCE-MRI. Differently from this study, however, we did not evaluate the performance of DBT and UE-MRI separately, since our aim was to highlight the potential of combining these two diagnostic methods to possibly counterbalance the weaknesses of both.

In our study UE-MRI+DBT had a higher underestimation rate of lesion size compared to DCE-MRI and a similar overestimation rate. Moreover, the evaluation performed with respect to histological subtypes showed better agreement measuring IDC both with UE-MRI+DBT and DCE-MRI. Differently from other studies (32, 33), no pure DCIS was missed, probably due to their large size (mean size = 28.5 mm) and to the presence of calcifications. It is known that UE-MRI has some limitations in the detection of DCIS, considering that the presence or absence of enhancement at DCE-MRI has been proposed as the preferable diagnostic criterion to rule out malignancy of mammographic calcifications (34), even if the underestimation of clearly suspicious calcification is never recommended, even in case of negative MRI (32). In these cases, DBT plays a fundamental role, since calcifications are easily recognizable and characterizable with this method. Therefore, the evaluation of UE-MR images after DBT could represent a way to overcome this major limit.

We also analyzed the potential for ADC values to predict lesions aggressiveness. Unlike previous studies (35–37), in our cohort there were no significant differences in terms of mean ADC values between immunohistochemical subtypes of breast cancer, but lower ADC values were associated with higher tumor grades. This result has confirmed evidences from previous studies (37, 38), suggesting that ADC values could predict nuclear grading of breast cancer.

Considering the aforementioned limitations of DCE-MRI, it would be desirable that new studies on faster, cheaper and unenhanced protocols could support the role of MRI both in the screening and the clinical setting. In this scenario, the aim of our work was not to subvert current international guidelines but to increase the knowledge about MRI and DBT and to provide new possible strategies in preoperative staging of breast cancer. Our preliminary results support UE-MRI as a valid alternative to DCE-MRI in women with a malignant lesion who had undergone DBT in the diagnostic phase, to reduce MRI protocol duration and costs, and to avoid the use of contrast medium.

Our study has some limitations. The analysis was monocentric and retrospective; our cohort was heterogeneous and limited in number; and we included in the study only patient with known malignant lesions, not considering a control group. Moreover, we did not evaluate the consistency level between DBT and UE-MRI in terms of lesion size agreement. Finally, IDCs were prevalent compared to other histological tumor types, even if it is well known that IDC is the most common type of breast cancer. However, our results are preliminary and data collection is still continuing.

## Conclusion

Our preliminary study demonstrated that both DCE-MRI and UE-MRI+DBT have good diagnostic performance for lesion detection and tumor size assessment in breast cancer patients. DCE-MRI confirmed to have the highest sensitivity, but UE-MRI+DBT showed a better accuracy and a slightly higher PPV, keeping a comparable sensitivity. Therefore, UE-MRI could be a valid alternative tool for preoperative staging when patients have already performed DBT to combine. Considering the limitations of this work, further multicenter, prospective studies will be useful to reach definitive conclusions.

## Data Availability Statement

The raw data supporting the conclusions of this article will be made available by the authors, without undue reservation.

## Ethics Statement

The studies involving human participants were reviewed and approved by Institutional review board. Written informed consent for participation was not required for this study in accordance with the national legislation and the institutional requirements.

## Author Contributions 

VR: Conceptualization, Methodology, Investigation, Data curation. GM: Investigation, Data curation. EK: Data curation. CC: Data curation. FP: Investigation, Data curation, Supervision, Validation. FG: Conceptualization, Investigation, Data curation, Supervision, Validation. All authors contributed to the article and approved the submitted version.

## Conflict of Interest

The authors declare that the research was conducted in the absence of any commercial or financial relationships that could be construed as a potential conflict of interest.
